# Supplementary material for: Self-supervised representation learning on gene expression data
Source: Bioinformatics. 2025 Oct 1;41(11):btaf533. doi: 10.1093/bioinformatics/btaf533 (PMC12611300; doi:10.1093/bioinformatics/btaf533)
Supplement: btaf533_Supplementary_Data [file btaf533_supplementary_data.pdf]

Self-supervised Representation Learning on Gene Expression Data: Supplementary Material

A Data Description

A.1 Preprocessing

To preprocess the ARCHS4 dataset, we first extract the different tissue types with the ARCHS4py Python package. We then normalize samples and correct for differences in gene count distribution before applying the batch-effect correction tool pyComBat, following the process described on the ARCHS4 platform. We applied the same normalization process to the TCGA dataset. The pre-processing was performed before splitting the datasets into pre-training and fine-tuning sets.

A.2 TCGA dataset description

	Size	Cancer	Non-cancer	Proportion (%)
Breast (BRCA)	1214	1101	113	12.9
Uterine (UCEC)	566	546	20	6.0
Kidney (KIRC)	597	534	68	6.3
Brain (LGG)	534	534	0	5.7
Head-neck (HNSC)	559	522	37	5.9
Lung (LUAD)	563	518	45	6.0
Thyroid (THCA)	513	513	0	5.4
Lung (LUSC)	517	501	17	5.5
Prostate (PRAD)	498	498	0	5.3
Skin (SKCM)	472	472	0	5.0
Lung (LOAD)	460	460	0	4.9
Ovary (OV)	429	429	0	4.5
Stomach (STAD)	462	412	50	4.9
Bladder (BLCA)	406	406	0	4.3
Liver (LIHC)	374	374	0	4.0
Cervix (CESC)	306	306	0	3.2
Kidney (KIRP)	321	291	30	3.4
Sarcoma (SARC)	263	263	0	2.8
Normal	289	0	289	3.0
Total	9349	8680	669	100

Table S1: Caracteristics of TCGA dataset

A.3 ARCHS4 dataset description

	Size	Cancer	Non-cancer	Proportion (%)
Brain	5463	661	4802	10.2
Skin	7506	255	7251	14.0
Neuron	2463	10	2453	4.6
Lung	6022	3840	2182	11.3
Colon	4531	1036	3495	8.5
Pancreas	868	253	615	1.6
Kidney	2695	337	2358	5.0
Heart	1197	0	1197	2.2
Liver	7515	1817	5698	14.1
Breast	7420	555	1865	13.9
Bladder	797	569	228	1.4
Cervix	390	64	326	0.7
Ovary	587	268	319	1.1
Prostate	2694	1675	1019	5.0
Sarcoma	1871	416	1455	3.5
Head-neck	19	1	18	0.03
Stomach	324	107	217	0.6
Thyroid	501	373	128	0.9
Uterine	419	39	380	0.7
Total	53282	15618	37664	100

Table S2: Characteristics of ARCHS4 dataset

B Pre-training Hyper-parameters

hyper-parameter	SCARF	VIME	BYOL
pre-training epochs	1000	500	50
batch size	256	32	32
optimizer	Adam	RMSprop	SGD
learning rate	10^{-4}	10^{-3}	10^{-4}
momentum	$\times$	$\times$	0.9
corruption rate c	0.3	0.3	0.3
temperature τ	1.0	$\times$	$\times$
trade-off α	$\times$	2.0	$\times$
decay rate λ	$\times$	$\times$	0.9

Table S3: Hyper-parameters for each methods.

C Fine-tuning hyper-parameters

hyper-parameter	Tested range	Best
Nb layers	[2,10]	4
Embedding size	{128,256,512,1024}	256
Batch Norm.	Yes/No	Yes
Dropout	[0.0, 0.5]	0.2
Optimizer	{SGD, RMSProp, Adam}	Adam
Learning rate	[1e−6, 1e−2]	1e−4
L1 regularization	[0, 1e−2]	1e−2
L2 regularization	[0, 1e−2]	1e−2
Batch size	[8,1024]	8

Table S4: Hyper-parameters tested.

D Architectures

D.1 Downstream task

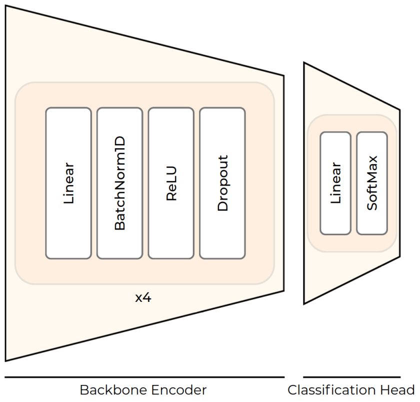

Figure S1: Fine-tuning step architecture

D.2 SCARF

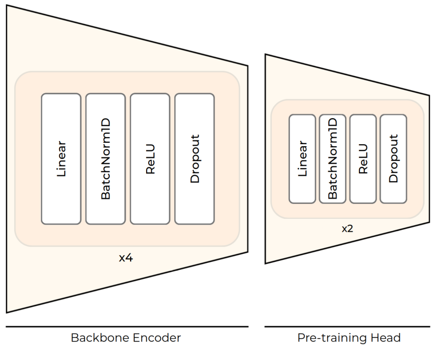

Figure S2: SCARF architecture

D.3 VIME

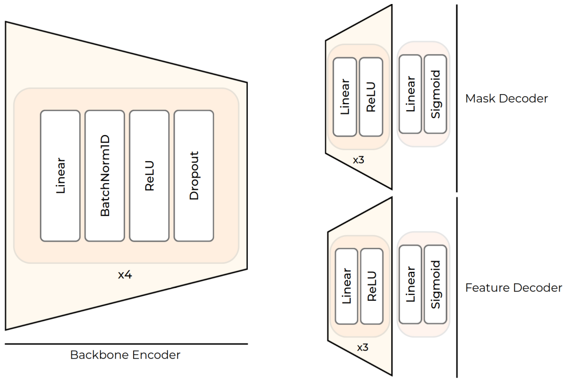

Figure S3: VIME architecture

D.4 BYOL

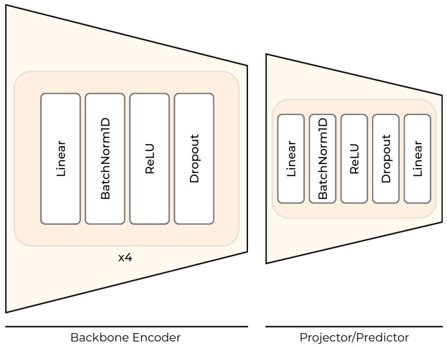

Figure S4: BYOL architecture

E Effect of augmentations/transformations

The following shows the effects of the two types of perturbation (uniform and empirical marginal) on the TCGA data. The results were obtained by applying these transformations to training data and minimizing cross-entropy loss to predict true labels. It shows that the applied transformations do not fundamentally perturb the underlying information contained in the examples.

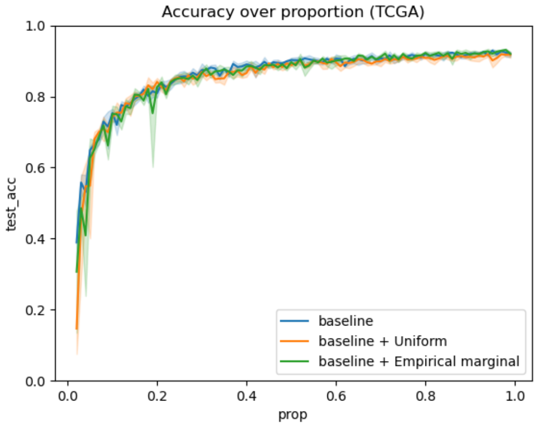

Figure S5: Effects of uniform and empirical marginal replacement on baseline accuracy

F Effect of augmentations on SCARF

The following shows the effect of the two types of transformation on the SCARF pre-training method. We compare the performance on downstream task between a SCARF model pre-trained with the uniform distribution replacement and another SCARF model pre-trained with the empirical marginal replacement. The baseline model trained from scratch is also provided as a point of comparison.

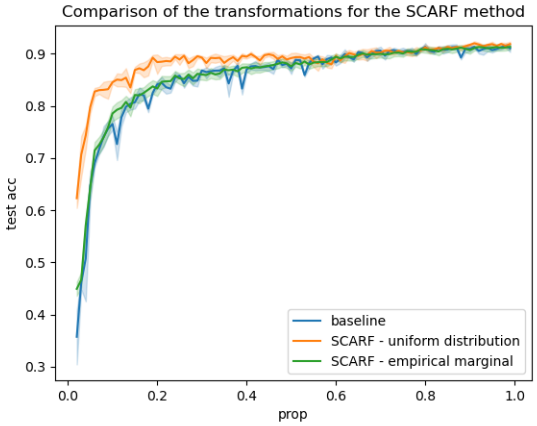

Figure S6: Comparison of the performance of SCARF on downstream task with the 2 type of transformation.

G BYOL loss

The following shows the development of the $\mathcal{L}_{\theta,\xi}$ loss, presented in Section 2.2.3.

$$
\begin{aligned}
\mathcal{L}_{\theta,\xi} &= \|\overline{q_\theta(z_\theta)} - \overline{\tilde{z}_\xi}\|_2^2 && (1)\\
&= \langle \overline{q_\theta(z_\theta)} - \overline{\tilde{z}_\xi}, \overline{q_\theta(z_\theta)} - \overline{\tilde{z}_\xi} \rangle && (2)\\
&= \langle \overline{q_\theta(z_\theta)}, \overline{q_\theta(z_\theta)} \rangle - 2\langle \overline{q_\theta(z_\theta)}, \overline{\tilde{z}_\xi} \rangle + \langle \overline{\tilde{z}_\xi}, \overline{\tilde{z}_\xi} \rangle && (3)\\
&= \|\overline{q_\theta(z_\theta)}\|_2^2 - 2\langle \overline{q_\theta(z_\theta)}, \overline{\tilde{z}_\xi} \rangle + \|\overline{\tilde{z}_\xi}\|_2^2 && (4)\\
&= 2 - \langle \overline{q_\theta(z_\theta)}, \overline{\tilde{z}_\xi} \rangle && (5)\\
&= 2 - 2\left\langle \frac{q_\theta(z_\theta)}{\|q_\theta(z_\theta)\|_2}, \frac{\tilde{z}_\xi}{\|\tilde{z}_\xi\|_2} \right\rangle && (6)\\
\mathcal{L}_{\theta,\xi} &= 2 - 2\frac{\langle q_\theta(z_\theta), \tilde{z}_\xi \rangle}{\|q_\theta(z_\theta)\|_2 \cdot \|\tilde{z}_\xi\|_2} && (7)
\end{aligned}
$$
